# CT Motion-Analysis of Implant Loosening in Total Wrist Arthroplasty: A Pilot Study

**DOI:** 10.1055/a-2528-0045

**Published:** 2025-02-21

**Authors:** Daniel Reiser, Sanj Kakar, Olof Sandberg, Per Wretenberg, Marcus Sagerfors

**Affiliations:** 1 Department of Orthopedics and Hand Surgery, Faculty of Medicine and Health, Örebro University, Örebro, Sweden; 2 Department of Orthopedic Surgery, Mayo Clinic, Rochester, Minnesota; 3 Sectra AB, Linköping, Sweden

**Keywords:** CT motion-analysis, implant loosening, total wrist arthroplasty

## Abstract

**Introduction:**

Total wrist arthroplasty (TWA) is a motion-preserving treatment option for wrist arthritis. High-precision measurement methods for implant migration such as computed tomography motion-analysis (CTMA) can potentially detect poor implant fixation. The aim of this pilot study was to assess CTMA as a complementary method to diagnose aseptic loosening of TWA.

**Materials and Methods:**

Three patients with a TWA and wrist pain during activity underwent induced displacement CT (CTMA) with alternated provocations as a complement to plain radiographs.

**Results:**

Two of the three patients had displacement of the carpal component on CTMA. The radial component was stable in all cases. The tool was adapted to clinical routine use.

**Conclusions:**

CT motion-analysis could be a valuable adjunct to plain radiographs in assessing component loosening in TWA.

Aseptic loosening is a critical issue in the long-term survival and failure of implants. Early implant migration serves as a significant indicator of potential implant failure. One precise method for measuring and visualizing implant migration in vivo is computed tomography-based radiostereometric analysis (CT RSA). This technique, developed in the early 2000s, leverages breakthroughs in computed tomography (CT) and computing technology, allowing high-resolution image acquisition at much lower radiation doses. Consequently, the measurement precision of CT RSA now rivals the current gold standard.

Total wrist arthroplasty (TWA) offers a motion-preserving alternative to arthrodesis for managing wrist arthritis and can give pain relief while preserving wrist motion. 1 Previous TWA designs have had issues with high revision rates. 2 Later TWA generations have shown improved survivorship and patient outcomes, but complications necessitating additional surgeries remain a substantial challenge. 3 A systematic review assessed TWA and wrist arthrodesis and found that while both TWA and wrist arthrodesis improve function, pain, and grip strength, there is a higher complication rate in TWA. 4 Implant loosening is the primary reason for revision surgery in TWA and loosening of the carpal component is more common compared to the radial component. 5

Plain radiographs can identify loosening through the presence of radiolucent lines (zones), but the findings can often be inconclusive. Substantial efforts have been made to develop methods for diagnosing aseptic loosening with higher accuracy. Nuclear medicine techniques like PET or SPECT-CT, can provide additional information, but due to normal bone activity, the results may be difficult to interpret. 6 Inducible displacement analysis offers the advantage of directly demonstrating and quantifying implant movement, rather than inferring its presence from secondary signs. This method has been a research focus since the 1990s, primarily for total hip arthroplasties (THA), using both radiostereometry analysis (RSA) and CT. 7 8 In the last decade, improvements in CT image quality, decreasing radiation doses, and computing power have made the inducible displacement method feasible for clinical practice (CT motion-analysis, CTMA).

To our knowledge, CTMA has not been used to assess implant loosening in TWA. The purpose of this pilot study was to assess CTMA as a complementary tool to plain radiographs in the assessment of implant loosening in TWA.

## Materials and Methods

This was a retrospective study. Eligible patients were those who had a CTMA of the wrist with a TWA at Örebro University Hospital in Sweden in 2024. The decision to have a CTMA was based on the treating surgeon deeming that there could be a value of added information to assess a TWA for aseptic loosening. Patients with TWA having wrist pain and clinically suspected implant loosening underwent anteroposterior and lateral wrist radiographs and CTMA of the wrist. Plain radiographs were taken according to clinical praxis, that is, anteroposterior and lateral views, and did not show periprosthetic osteolysis (PPO) or zones around the components for two of the patients. Lab tests were taken (ESR and CRP). Two of the patients had a newly developed TWA with a coating of tantalum and a metal-and-polyethylene articulation developed in collaboration with TriMed Inc. which is not commercially available, and one had a Biax TWA (DePuy Orthopedics, Warzaw, Indiana, USA, now discontinued). The mid-term outcome for a previous version (using Hydroxyapatite coating and an articular liner of Carbon Fiber Reinforced PolyEther-Ether Ketone, CFR-PEEK) of the newly developed TWA has been reported. 9

**Fig. 1:**
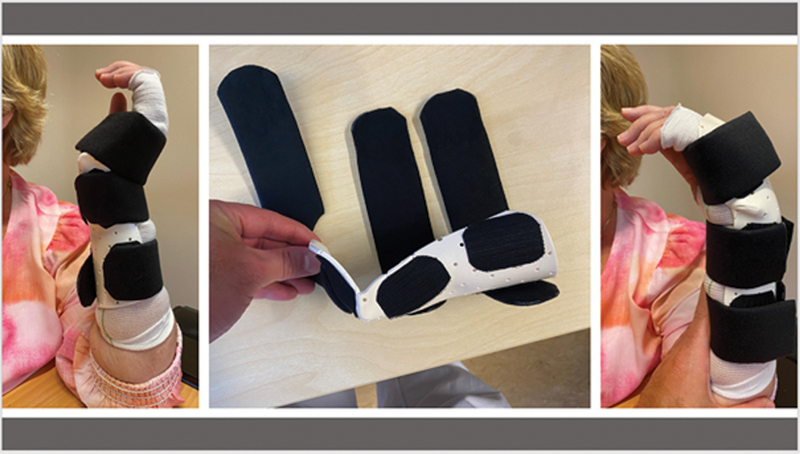
Splint for CTMA-induced displacement wrist extension and flexion.

A low-dose CT protocol was created by adapting recommended settings to the local equipment, a Siemens Somatom Definition AS (64 slice). The settings on the CT were 120 kV, 100 ref mAs, slice thickness of 0.6 mm, increment 0.6 mm, pitch 1.0, and rotation time of 1.0 seconds. For each scan, 1 axial series with and 1 without iMAR (iterative metal artifact reduction) was saved. The wrist was examined in two positions, maximum flexion, and maximum extension. Wrist orthoses were made in advance for maximum flexion/extension so that the patient could hold the wrist in maximum flexion or extension during the CTMA examination ( Fig. 1 ). The radiation dose of this protocol was about 0.01 mSv per scan—as a reference, 0.5–1 mSv represents the annual background radiation in Sweden. The method measures the movement of the radial stem against the radius and the carpal component against the third metacarpal and the capitate. The study was approved by the Swedish Ethical Review Authority (no. 2024-05710-01).

## Results

**Fig. 2:**
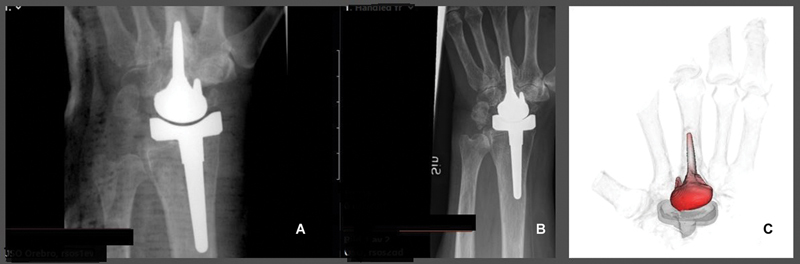
Patient 1; ( **A** ) postoperative radiographs; ( **B** ) latest radiographs; ( **C** ) CTMA; The red part indicates movement of 3.6 mm and 7.7 degree of the implant versus the third metacarpal.

Three patients were included. The first patient was a 74-year-old female with a Biax-TWA due to an underlying rheumatic disease who, 20 years after the initial TWA procedure, developed clinical symptoms and also showed some radiographic signs of loosening of the carpal component. Whether there was loosening of the radial component was difficult to assess. The CTMA showed substantial motion of the distal component during the inducible displacement, indicating loosening. The movement of the radial stem was measured against the radius and the carpal component versus the third metacarpal. The CTMA showed 3.6 mm and 7.7 degree movement of the carpal component versus the third metacarpal and no movement of the radial component versus the radius ( Fig. 2 ).

**Fig. 3:**
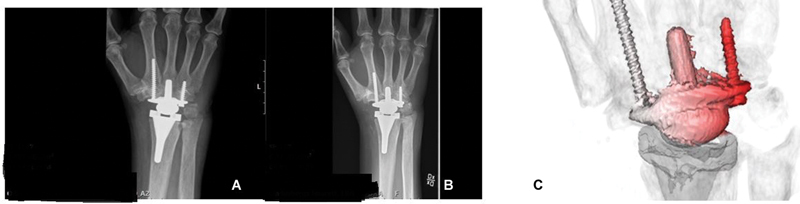
Patient 2; ( **A** ) postoperative radiographs; ( **B** ) latest radiographs 2024; ( **C** ) CTMA; In this case, the reddest area moves 0.16 mm and 0.6 degree relative to the third metacarpal.

The second patient was a 73-year-old male with an underlying rheumatic disease with the new TWA design had some clinical wrist symptoms like pain during activity for the last 6 months. The index operation was 8 years earlier and the patients came for a planned follow-up visit. The plain radiographs did not show any signs of implant loosening but the CTMA with inducible displacement showed loosening ( Fig. 3 ). In this case, the reddest area of the carpal component moved 0.16 mm and 0.6 degrees relative to the third metacarpal. The radial component showed no signs of loosening ( Fig. 3 ).

**Fig. 4:**
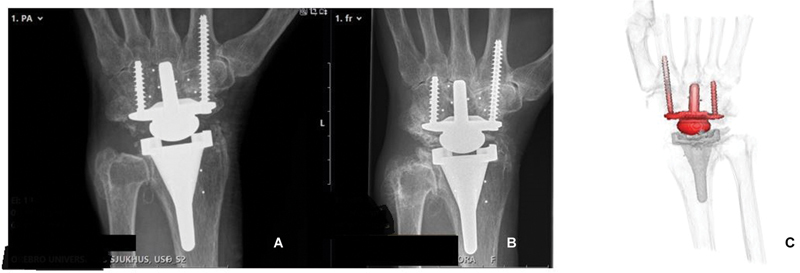
Patient 3; ( **A** ) postoperative radiographs; ( **B** ) latest radiographs; ( **C** ) CTMA; In this case, the reddest area moved 0.7 mm and 1.5 degree relative to the third metacarpal.

The third patient was a 70-year-old woman with an underlying rheumatic disease and low functional demands who also had a TWA with the new design with tantalum coating on both the radial and carpal components and came for a scheduled 8-year follow-up with some wrist pain during activity. She had no plain radiographic signs of loosening. CTMA did detect movement of the carpal component. In this wrist, the movement was assessed of the radial stem against the radius and the carpal component against the third metacarpal and the capitate. The carpal component had a movement of 0.7 mm and 1.5 degree relative to the 3rd metacarpal ( Fig. 4 ). For all patients, the lab tests (ESR and CRP) were normal.

## Discussion

The findings from this pilot study indicate that CTMA with induced displacement can detect aseptic loosening of a TWA despite normal plain radiographs. This displacement CT method can demonstrate the movement of a loose component via visualization and high-precision quantification in 3D. Instead of relying on secondary signs like osteolysis or uptake of radioactive compounds, the mechanical instability itself is visualized. Like a regular static CT, the displacement CTMA can also include a description of secondary signs like osteolytic zones.

TWA is an established motion-preserving alternative to arthrodesis in wrist arthritis. Previous TWA designs have demonstrated not only improved range of motion (ROM) and reduced pain scores but also high revision rates. 10 Improved survivorship and patient-reported outcomes have been noted with contemporary designs, but complications after TWA requiring additional surgery remain an issue. 11 One of the main challenges with TWA is loosening. Implant loosening can sometimes be diagnosed clinically and with plain radiographs. On plain radiography, loosening can be detected as bone destruction in the form of radiolucent lines (zones) or PPO but in some cases the results from plain radiography are inconclusive. In addition, PPO is not always associated with implant loosening. 12 Substantial efforts have been put into developing methods to diagnose aseptic loosening with higher accuracy. 6 13

Two of the TWAs in this study were a later version of a previously reported TWA design. 9 The third TWA studied was a Biax. The patient with the Biax TWA showed signs of loosening in the third metacarpal both clinically and radiographically. The risk of the carpal component of the Biax TWA penetrating into the third metacarpal has been noted in a previous paper. 14 The patient with clinical complaints 6 months before the CTMA showed no signs of loosening radiographically, but the CTMA demonstrated loosening of the carpal component. In our experience, the qualitative assessment of the loosening is probably a better indicator of implant loosening compared to a strict quantification. In addition, a strictly quantitative assessment can be cumbersome as it is presently unknown how many 10ths of a millimeter represents a clinically relevant cut-off for aseptic loosening. The fact that the radial component was stable is encouraging but underlines that the carpal fixation of a TWA remains problematic. Establishing the diagnosis of implant loosening in TWA is based on a combination of medical history, a thorough clinical examination, and imaging diagnostics. Revision to another TWA can be possible but the outcome can be unpredictable and as many as 25% require additional surgery. 15 Revision to a radiocarpal arthrodesis (RCA) is another option but the outcome is inferior compared to a primary RCA. 16 The findings in this pilot study indicated TWA loosening despite no apparent signs on plain radiographs. Whether or not to proceed with a revision of the TWA is part of a shared decision-making process between the patient and the hand surgeon. The patients with loosening were offered revision surgery but declined surgery at this time due to other health problems unrelated to the wrist.

This method has previously been shown in Total Hip and Knee Arthroplasty to be able to differentiate true movement from noise with a precision level of around 0.1–0.3 mm. 17 18 A recent study on shoulder arthroplasty found that CTMA can be used to identify early migration and the development of radiolucent lines over time in glenoid components. 19 The CTMA technique has also been used to study the migration and micromotion of fracture fragments after operative treatment of distal radius fractures and pelvic fractures. 20 21 How this translates to TWA remains to be studied. However, we believe it is important to differentiate between this precision level, which indicates how large a movement must be in order to reliably rise above the noise level, and the amount of movement that is required for an implant to have what can be considered a clinically relevant degree of loosening. This latter level is likely more complex and variable between patients. This would indicate that setting a threshold level for what degree of movement that should be considered clinically relevant can be challenging. It is interesting to note that, in our opinion, no clear boundary can be drawn between normal micro-movement of the TWA and pathological, clinically relevant loosening. The second patient had 0.16 mm and 0.6 degree movement of the component relative to the third metacarpal and clinical loosening signs; the third patient, who had no definitive clinical loosening signs, had a component movement of 0.7 mm and 1.5 degree relative to the capitate. The third patient is an elderly lady with low functional demands on her hands. The second patient, despite his age, still works part-time as a craftsman and mainly has load-related pain. We speculate that the boundary between clinically relevant and irrelevant movement of the TWA is probably influenced by various factors, including the patient's load profile. The CTMA comes with an additional cost, but could potentially identify implant loosening at an earlier stage. This could enable an earlier correct diagnosis of implant loosening which could avoid massive loosening and bone loss. A quick correct diagnosis could also minimize sick leave from work.

**Fig. 5:**
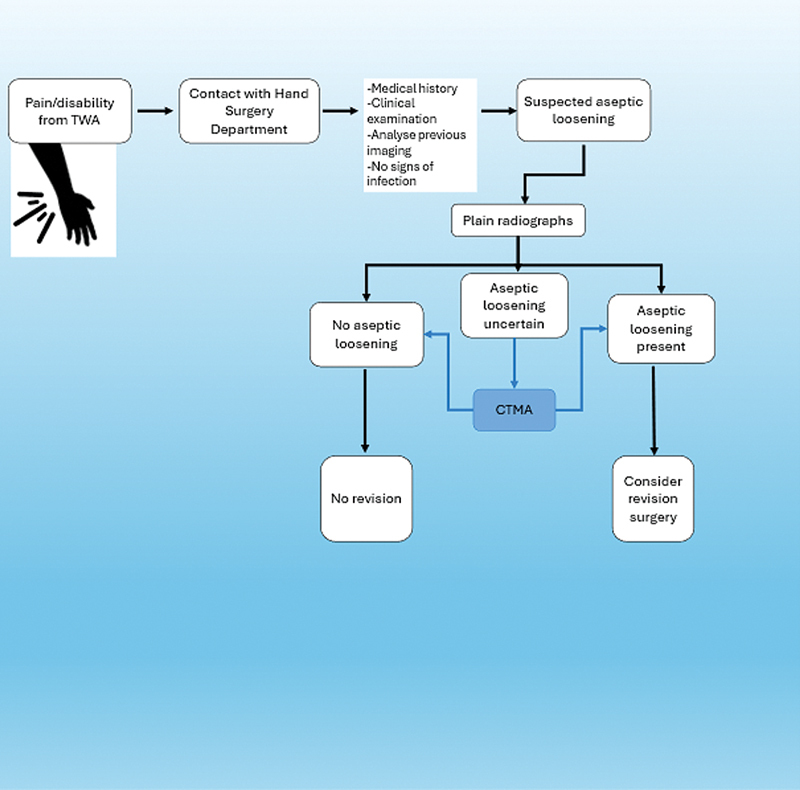
Proposed flow chart.

We propose an improved patient flow where patients with pain during activity from a TWA wrist and with no radiographic loosening can benefit from an inducible movement CTMA for improved diagnostic accuracy ( Fig. 5 ).

One limitation is whether a reliable mode of provocation can be determined if the patient cannot comply with the provocation protocol, too small a provocation can make it hard to draw conclusions. It is known that reduced ROM and tremors can reduce the possibility to detect implant loosening using CTMA. 22 It can be argued that if revision of the TWA is not an option, displacement CTMA should not be done. However, a diagnosis of a loose TWA component may eliminate the need to pursue other causes for the patient’s symptoms. Another limitation of the study is that the TWAs used in this pilot study are not commercially available today. However, the TWAs in this study share design features with other TWA designs, and the results are probably applicable to other TWAs. The small number of patients is also a limitation. Larger studies, preferably using different TWA designs, are warranted to assess the correlation between clinical symptoms, plain radiographs, and micromotion detected on CTMA. This could potentially help establish a clinically relevant cut-off for aseptic loosening.

## Conclusion

The results of this pilot study indicate that CTMA with inducible displacement could be a valuable adjunct to plain radiographs in assessing implant loosening in TWA.
